# Serious Motion-Based Exercise Games for Older Adults: Evaluation of Usability, Performance, and Pain Mitigation

**DOI:** 10.2196/14182

**Published:** 2020-04-01

**Authors:** Philipp Brauner, Martina Ziefle

**Affiliations:** 1 Human-Computer-Interaction Center RWTH Aachen University Aachen Germany

**Keywords:** serious games, exercise game, health care, pain, ambient assisted living, technology acceptance

## Abstract

**Background:**

Many societies are facing demographic changes that challenge the viability of health and welfare systems. Serious games for health care and ambient assisted living (AAL) offer health benefits and support for older adults and may mitigate some of the negative effects of the demographic shift.

**Objective:**

This study aimed to examine the acceptance of serious games to promote physical health in AAL environments. Since AAL environments are designed specifically to support independent living in older adults, we studied the relationship among age and user diversity, performance in the game, and overall usability and acceptance evaluation.

**Methods:**

We developed a motion-based serious exercise game for prototypical AAL environments. In two evaluations, outside (n=71) and within (n=64) the AAL environment, we investigated the influence of age, gender, self-efficacy in interacting with technology, need for achievement on performance, effect of the game, usability evaluation of the game, and overall acceptance.

**Results:**

Both games were evaluated as easy to use and fun to play. Both game interventions had a strong pain-mitigating effect in older adults (game 1: −55%, *P*=.002; game 2: −66%, *P*=.01).

**Conclusions:**

Serious exercise games outside and inside AAL environments can contribute to individuals’ health and well-being and to the stability of health care systems.

## Introduction

### Background

Many societies are facing a demographic shift because of declining birthrates and increased life expectancy [1]. It is estimated that the proportion of people 65 years and older will increase from 17% as recorded in 2008 to an estimated 30% by 2060, and that the proportion of those 80 years and older will almost triple, from 5% to 14% [2], in the same time period. Given that the risk of acute and chronic illnesses increases with age [3-6], this means a shrinking workforce will be financially responsible for a growing number of older adults with ever greater medical and general care needs.

Two *possible* solutions to address these challenges are the *ambient assisted living* (AAL) *environments* and *serious games for health care*. AAL supports people in need of care through technology that can compensate for health-related restrictions, while increasing comfort and safety [7-10]. Serious games combine elements of play with serious goals, such as learning or exercising [11-14].

A challenge while designing information and communication technology–based health interventions is age-inclusiveness: Older adults often report lower performance and self-efficacy levels in their interaction with digital interfaces [15,16]. In addition, age-related physical limitations in motor ability, cognition, and visual acuity are further usage barriers [17,18]. In addition, it is possible that older adults may have different ideas about the content and style of games than those proposed by typically younger game designers [19-21]. Nevertheless, despite the common misconception that older adults are averse to new technology, most are open to innovation and willing to master new technologies—so long as they are comprehensible, aligned with their values, and address their wants and needs [22-24]. Consequently, there is a demand for helpful, easy to use, and well-designed assistive and autonomy-enhancing technologies. However, their success may well depend on being designed, developed, and evaluated with and by the target group, older adults.

This paper presents the iterative development of a serious game for older adults to increase physical fitness in the AAL environments, including two user studies. The paper begins by presenting background on aging, the effects of exercise, the benefits of serious games in health care, and AAL, as well as our research objectives. Next, we describe the design of the two game iterations, our experimental approach, and the participants. We then present the experimental findings of the participants’ performance, the impact of the games, and an evaluation of the game interface usability. We end by discussing the results and their implications, future research questions, and limitations of this work.

### Age, Health, and the Benefits of Exercise for Older Adults

Aging is associated with an increased likelihood of chronic illnesses or disabilities, such as mild cognitive impairment, dementia, or Alzheimer disease; ischemic heart disease; congestive heart failure, stroke, and diabetes mellitus [3,6,25,26].

Physical exercise can increase overall health and well-being, and it can reduce the risk of illnesses. Although exercise intensity, frequency, and duration can always be optimized, some activity is always considered better than none [27]. Only 150 min of medium-intensity exercise per week can produce positive effects on health and are recommended, in particular, for children, overweight people, and older adults [28] by the World Health Organization [29]. A combination of regular and life-long aerobic activities and resistance, flexibility, and balance exercises is suggested for people suffering from chronic syndromes [30].

The benefits of exercise are manifold. Strength training mitigates age-related decline in muscle mass, strength, and performance [31]. In addition, exercise can also reduce the probability of silent brain infarcts by 40% [32], has a positive influence on migraines [33], can mitigate the symptoms of depression [34], and can reduce drug abuse among the elderly [35]. It also improves executive function in healthy people [36,37], reduces the symptoms of mild cognitive impairment [38], and reduces the risk of mild cognitive impairment and dementia in later life [39].

However, despite its many benefits, physical activity is decreasing in North America, Europe, and many other countries [40]. Recent medical reports have shown higher incidences of hypertension, diabetes, and coronary or cerebrovascular diseases, and overall lower life expectancy [41,42]. These findings indicate that increasing physical activity—especially in older adults—merits serious attention, along with developing a better understanding of how to motivate people to engage in more frequent exercise.

### Serious Games for Health Care and Design Guidelines

Serious games combine the motivational attraction of games in general and computer games in particular with serious activities and outcomes through play. They build on the Premack principle [43], that is, the likelihood of performing unpleasant activities—such as training or exercise—increases by linking them with pleasant activities—such as playing games.

Various scholars have defined the concept of serious games. An early definition by Abt [11] describes serious games as having “... an explicit and carefully thought-out educational purpose and are not intended to be played primarily for amusement.”. A similar definition stems from Michael and Chen [44], who defined them as “a game in which education (in its various forms) is the primary goal, rather than entertainment.”. Bogost [12], in contrast, prefers the term *persuasive games* for games that use “procedural rhetoric to support or challenge our understanding of the way things in the world do or should work.”

That said, the field of serious games, in general, and serious games for health care, in particular, is vast. As such, the following paragraphs seek to provide only a broad overview of the state of knowledge of the effects of serious games in health care.

As early as 1990, Whitcomb [45] studied computer games for older adults and argued that appropriately designed games increased reaction times, eye-hand coordination, and dexterity. He claimed that these gains were transferable and could also have real-life benefits, but he was critical that older adults appeared to enjoy few games, likely because of inappropriate sounds or speed, or poor visibility of the game elements.

King et al [46] developed a game-based system for patients with stroke to retrain movement abilities of the upper limbs. The rehabilitation task involved selecting on-screen objects with speed and accuracy using a gyroscope-based controller. Despite limited direct effects on motor performance, the game was found to be motivating, and it increased rehabilitation adherence.

Flores et al [47] found that patients perceived robotic rehabilitation systems as boring, and this reduced rehabilitation motivation and adherence. They identified a gap between available rehabilitation and commercial game titles. Although the latter were more entertaining, they were not adaptable to rehabilitation tasks. Rehabilitation games, in contrast, were usually developed by engineers and medical professionals with a strong focus on task fit, and they often lacked motivating play.

Gabrielli et al [48] evaluated several low-cost gaming platforms—such as Microsoft Kinect, Stifteo Cubes, and Simon’s game—as rehabilitation instruments for patients with stroke. Although the systems were considered effective, the participants expressed a desire for greater accessibility, usability, and more motivating game play.

Several minigames for balance training in older adults were designed for the *Nintendo Wii Balance Board* [49]. Their evaluation underscored the importance of considering age-related and individual constraints when developing motivating and usable serious games in health care. Further research supported the claim that computer-mediated balance training increased mobility in older adults and—as a side effect—the multitasking abilities of the participants [50].

Serious games can also convey knowledge and improve the ability to cope with the disease. For instance, Fuchsloher et al [51] developed games for diabetes management in teenagers. They found that the game variant that explicitly featured diabetes content was perceived as more enjoyable than the variant which conveyed the same learning objective content albeit implicitly. Further, a meta-review of motion-based video games found that most motion-based video games yielded sufficient activity levels to meet the health and fitness guidelines [52].

### Guidelines for the Design of Exercise Games

Older adults and chronically ill people have specific demands that must be considered when designing applications and serious games. These constraints include changing interests or values [19] over the life span, age-related acuity loss, motor speed and accuracy, restrictions on mobility, lower information-processing speed, and lower reaction times [17,18]. Consequently, contents, mechanics, and interfaces of serious games for health care should be tailored to the target audience.

Several guidelines facilitating the systematic development and customization of serious games for health care have emerged over the years. Weismann [53] studied the suitability of computer games for older adults in the 1980s. The guidelines he derived remain relevant. For instance, he suggested that games should be simple, and the next steps and the overall goals should be transparent. The visual complexity of the game should be appropriate, for example, by avoiding too many, too fast, or unrecognizable screen objects and symbols. Sound effects should be provided as auditory feedback, for rewards, or as cues that are clear and distinct. Also, control of the game character(s) should use natural mappings. The guideline also suggested that personalization by naming game characters and playing with peers increased the game’s entertainment value.

Ijsselsteijn et al [54] added further requirements for older adults. They proposed that games should have visual settings that are easy to adjust to individual visual changes. They suggested that information should be given redundantly, for example, by using multiple output modalities (eg, combine visual feedback with auditory feedback). In addition, memory load and required cognitive processing should be kept low to deal with age-related cognitive decline (eg, remembering information from one screen to another should be avoided). Also, that the game should provide enough time to learn and rehearse the necessary skills. Finally, the authors suggested that to overcome the potential anxiety of some players, encouraging feedback should be provided from the outset.

Whitehead et al [55] formulated guidelines specifically for exercise games. For instance, they proposed that the game should monitor the correct execution of the intended body movements and incorrect or intentionally forged movements should not be accepted by the game (anecdotally, often observed in younger *Wii* players). They suggested that activities within the game should focus on larger muscles, such as the arm or leg gestures. Also, that getting experience in the game should be rewarded, as higher experience appeared to relate to higher physical benefits. Rewards should also incentivize long-term use, as continuous and long-term usage has more benefits than short and singular activity bursts. As exercise games can be exhausting, the games should also include recovery times between the active phases. Most importantly, the exercise game should provide an abstraction from the physical activity or health care context and must transfer the exercises in another, more playful, scenario.

Although the previous guideline involved general exercise games for health care, Gerling et al [17] developed seven recommendations specifically for exercise games for the elderly. They are summarized as follows: First, games should account for potential impairments and reduced abilities of the players. Second, they should be adaptable to individual limitations in the range of motion that players might have. Third, the games should alternate between physically demanding and more relaxing tasks or even breaks to prevent overexertion. Forth, to address varying player abilities, the games should dynamically adjust difficulty to provide an appropriate level of activity and challenge. Fifth, the game should provide clear instructions for gestures, and these should be intuitive or easy to learn and relate to real-world activities. Sixth, tutorials and hints should guide the players through the game and its tasks, and it avoid the feeling of being lost. Seventh, the game should be easy to set up and run, with complicated setup menus and calibration processes avoided.

### Ambient Assisted Living

AAL is the seamless integration of sensors, actuators, and communication technology in the physical surrounding to enhance safety, quality of life, and independence in older adults [7,10,56,57] with the goal of facilitating *aging in place* [58]. Examples of AAL technologies include invisibly integrated sensors for fall detection, systems supporting the punctual and well-dosed intake of medication, communication channels to family or physicians, and assistive robots as supporting social actors [59-61]. The key, however, is ensuring that technology for aging in place is aligned with older adults’ needs and wishes and, most importantly, for usage motivation that they accept it [62,63].

### Objective and Aim of the Study

Although the AAL technologies are becoming a reality, they are entering our lives quite slowly. Neither their challenges and opportunities nor the wants and needs of future residents have been sufficiently studied [23,61,64-66]. This research gap is problematic as the perception and use of technology differ individually and are influenced by factors such as age, gender, chronic illnesses, or competence beliefs [15,16,23,66]. Therefore, this paper addresses the following aspect:

1. We present the iterative and participatory design process of a motion-based exercise game for older adults and its embedding into a prototypic AAL environment.
2. We analyze if and how user diversity influences the performance attained in the game, its impact on usability evaluation, and its relationship with the overall acceptance of the system. We specifically focus on the effect of age, as age is often linked to lower performance, perceived usefulness, and ease of use, as well as lower overall acceptance (see above).
3. As part of an exploratory study, we show that interacting with the game might have a pain-mitigating effect in older adults.

## Methods

### Overview

We present two studies to evaluate the effect and perception of serious games for health care. This section describes our AAL lab; the two game demonstrators outside and within the AAL environment; our experimental approach to study performance, acceptance, and effect of the games; as well as samples of both studies.

### An Exploratory Lab for Ambient Assisted Living

In the above section, we introduced AAL as a technology-based approach to increase the autonomy, safety, and comfort of older adults or people with chronic illnesses. Many of these concept technologies are still under development and not readily available. To study how older adults interact with these novel technology-augmented habitats, whether they accept the tight integration of technology in their life, and how to tailor technology to their wants and needs, prototypic assistive technology was integrated and tested in living labs.

One such type of these prototypical living environments is the AAL lab located at Rheinisch-Westfälische Technische Hochschule Aachen University. It resembles a living room of approximately 25 m^2^, with couches, a table, shelves, and lamps, and pictures on the walls. It was designed for patients with an artificial heart who might require tight monitoring of their vital signs, such as weight, body temperature, coagulation, and blood pressure [8]. As such, numerous sensors and actuators are invisibly integrated into the surrounding. When desired, a scale integrated unnoticeably in the parquet measures the weight of the residents, sensors in the floor can detect falls and request aid, and an invisible infrared camera detects possible infections from a distance by measuring body temperature [8]. In addition, several surfaces in the room serve as interactive media for prototyping and evaluating new applications, such as telemedical consultations on a large multitouch wall. More information about the prototypic lab can be accessed over the Web [67].

### Realized Game Prototypes

Our goal was to augment the functional aspects of the AAL environment with hedonic and playful, yet medically useful activities. Therefore, we designed a motion-based exercise game for this living room using an iterative, user-centered, and participatory design process. As an intermediate step, we developed and evaluated a stand-alone exercise game and then adapted the game based on feedback to the AAL environment. Both games and their evaluations are presented in the following sections.

The first stand-alone motion-based exercise game was designed to be set up in a living room, a doctor’s office, or in a retirement home. The second demonstrator is tightly integrated into the AAL lab and uses the input and output devices seamlessly embedded in the environment. Both games use a gardening scenario where the player must perform various movement gestures to collect different fruits and place them in a basket. Each fruit is linked to different gestures developed in collaboration with orthopedists and physiotherapists (details differed between the games, see below).

A Microsoft Kinect sensor tracks the player’s position and body posture though a skeleton model. The player sees a representation of themselves in the garden environment, either as a virtual avatar in the first prototype or as a background-separated video image in the second prototype. Both games are loosely based on the game *GrabApple* that also uses a garden environment and fruit collection to stimulate movements for office workers [68]. The design and development process used the guidelines presented above [17,18,20,53,69]. Design decisions were assessed regularly. We paid particular attention to age-inclusive design, for example, using large and contrast-rich game elements and clear auditory feedback.

#### Physical Exercise Game Outside the Ambient Assisted Living Environment (Game 1)

We used the Unity game engine to build the first functional game prototype. A Microsoft Kinect sensor captured the players’ body pose with 20 edges and mapped these to a virtual avatar presented in the garden environments. Players were instructed to collect different fruits and vegetables using movement gestures introduced over the course of the game (see Multimedia Appendix 1):

- The first level consisted only of Apples. These appeared on trees located in the garden and players collected them by moving one hand to the apple and then to a basket.
- The second level introduced Carrots as an additional object. In contrast to the apples, these appeared on the ground and could be collected by bending over and picking them up with the hand.
- The last level introduced Bananas as a third category. This level required moving one hand to the target, holding that posture for a short time (≥500 ms), and then placing the banana in the container.

**Figure 1:**
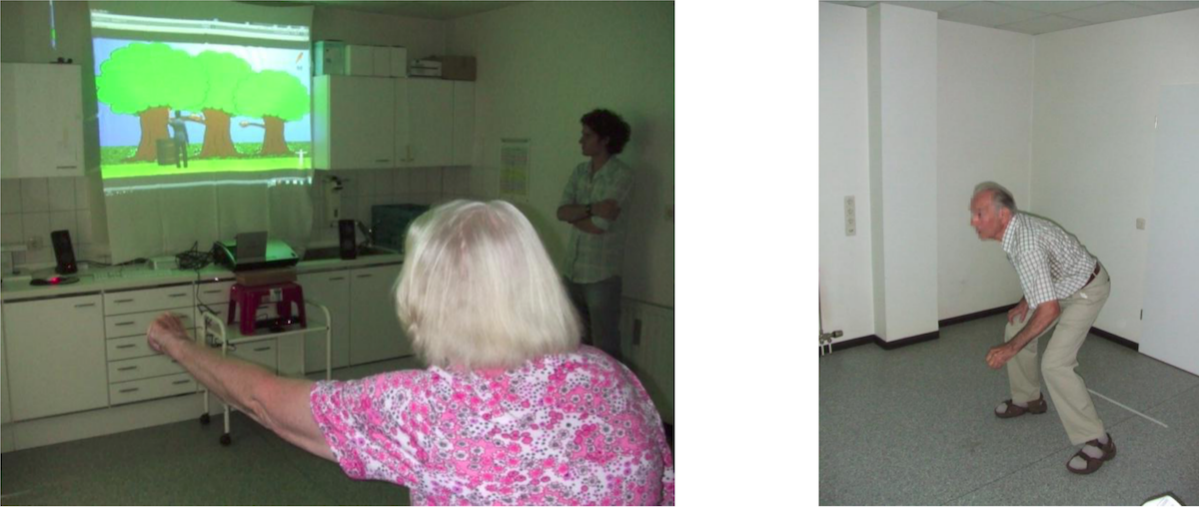
Two players of the first game prototype in a doctor’s office grabbing an apple (left) and bending for a banana (right).

More targets with additional movement gestures were implemented, but they were not part of the subsequent evaluation. Figure 1 shows two older persons interacting with the game.

A challenge while building motion-based exercise games is to ensure that all players can reach all required positions and perform all gestures. Thus, a calibration screen at the start of the game collected information about the participant’s body height and range of motion by asking the player to reach out to specific positions. The game then scaled the environment to the player’s range of motion and ensured that the player could reach the game objects with similar effort.

#### Physical Exercise Game for the Ambient Assisted Living Environment (Game 2)

**Figure 2:**
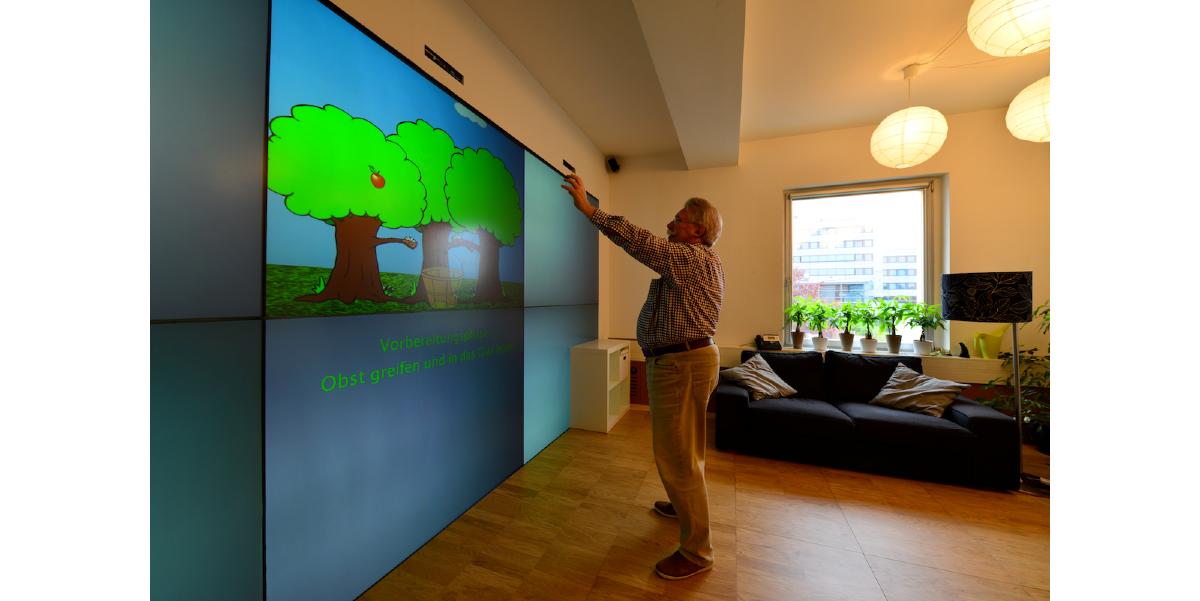
Player interacting with the exercise game in the ambient assisted living environment.

Using feedback from the first game’s evaluation, the second demonstrator was then integrated in the AAL environment and adapted given the constraints of the room (see Figure 2). For instance, as the Kinect Sensors were located near the ceiling, we temporarily removed the carrots (*bending + grabbing* gesture) and only used gestures performable in an upright pose (see Multimedia Appendix 1):

- Again, the game started with Apples in the first level. As with the first game, the player could collect apples by moving one hand to the apple and then moving the hand to a basket.
- Then the game introduced Bananas that required touching the object for a short time and then placing the fruit in the basket.
- The third level introduced Pears requiring a diagonal movement, that is, if they appeared on the right, they were grabbed with the left hand.

Reducing the gesture set because of technical constraints was obviously regrettable. However, we believe participants can still evaluate the game, and that foreseeable technical progress will make the sensors smaller and better integrated into the environment.

We also improved upon many issues observed when participants interacted with the first game. Three key aspects are illustrated below:

- Although the test persons found the calibration screen easy to use, they described it as unnecessary and not well integrated. We, therefore, replaced it with an implicit calibration mechanism. Through smart placement of the first-game objects and continuous measurement of the interactions, the user-adaptive scaling of the game environment can now be used from the beginning of gameplay.
- Despite a visual timer, some players reported difficulties keeping track of time in a given level when they became absorbed in the game. Thus, we changed the static horizon in the background and let it cycle through the day from midday sun to sunset. The participants found this method easier to comprehend, as they intuitively understood the day’s end mapped to the end of the level.
- We frequently observed that players left the detection area of the Kinect sensor. The players disliked the textual hints or eventual error messages in the first game, as these disrupted the immersion. We addressed this problem by introducing clouds (and eventually lightning and thunderstorms) when the player moved too far to the left or right, thus helping to nudge the players back to the center.

Multimedia Appendix 2 presents the iterative design process of the game by showing the different research objectives at each development stage, the methods used, and the key insights addressed in later iterations. Multimedia Appendix 1 shows the gesture sets for both games.

### Evaluation Framework

This section presents the framework we used to evaluate both game prototypes. We present the user factors, the within-subject variables (repeated measures), and the target variable used to evaluate the usability and social acceptance of the game.

We began the session by welcoming the participants to the study in the doctor’s office or our living lab, offered beverages, gave an introduction to the study. This gave participants time to rest and become comfortable in the test setting. Next, we surveyed the participants on user factors.

#### User Factors

User factors were as follows:

- Age in years: We requested the participant’s age to study its influence on performance, game evaluation, and projected acceptance.
- Gender: Gender is linked to usage, perceived ease of use, and self-efficacy in interacting with information and communication technology [70]. Thus, we wanted to evaluate whether gender also influenced game evaluation (dummy coded as male=1, female=2).
- Self-efficacy in interacting with technology (SET): Self-efficacy is the domain-specific belief, rooted in the perception of one's own competence, that one can be successful in a certain activity [71] and SET is usually correlated with age and gender on the one hand and determines our performance and our experience of competence in interacting with new technology on the other [15,70]. Consequently, we measured the participants’ self-efficacy when interacting with technology using four 6-point Likert items on a scale developed by Beier [72] with internal reliability of alpha≥.830.
- Need for Achievement (NfA): We assume that a person’s NfA is related to the attained performance in the games and wanted to evaluate if the NfA or the attained performance was related to acceptance. NfA was measured on six items on a 6-point Likert scale developed by Schuler [73]. The scale has internal reliability of alpha≥.899.
- Gaming frequency (GF): The participants were asked for their current GF across multiple games, game domains, and media, such as games of skill, board, and card games, and also games mediated through game consoles and mobile phones, as well as ball and outdoor games. The scale has internal reliability of alpha≥.730 and is strongly correlated with the single item “I enjoy playing games” (*r*=0.558, *P*<.001).

#### Repeated Measures Variables

We measured perceived pain (PAIN) and perceived exertion directly before and directly after the game, as well as attained performance across three levels of the games:

- PAIN: The participants reported their perceived level of pain for eight parts of the body on a 6-point scale before and after playing. The range was no pain to severe pain [74]. The scale measured reliability at alpha≥.786.
- Perceived exertion: We used a single-item scale developed by Borg [75] to assess perceived exertion just before and immediately after the game intervention. We did not measure heart rate directly so as to minimize stress in the participants, as some might feel uncomfortable with hard medical measures during the study. However, Borg scale has been found to be strongly correlated to actual heart rate [75].
- Performance: The players’ performance in the game was measured using log files as collected objects per minute in the first, second, and third levels of the game. However, note that performance was not directly comparable across the games, as they differed slightly and required different movement gestures.

#### Dependent Variables

**Figure 3:**
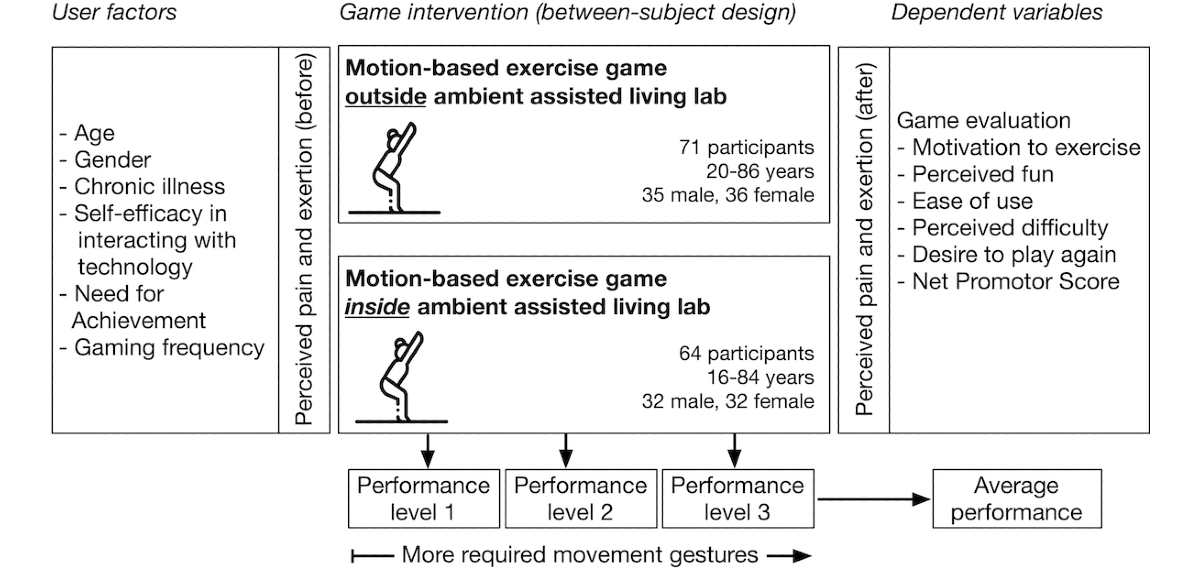
Experimental framework for evaluating the impact and usability of both game prototypes.

Dependent variables were as follows:

- Summative evaluation: The players of both games were asked to evaluate perceived fun, if they wanted to play the game again, or if they wanted to play these games in their home environment. In regard to the overall usability, we assessed whether participants were satisfied with the visual presentation, the visual and auditory feedback, if they understood the game concept, and if they felt in control while using the motion-based gestures. In addition, we asked if the game’s overall difficulty and the difficulty of performing the gestures were well adjusted. These questions were measured on 6-point Likert scales.
- Net promoter score (NPS): Finally, we asked how likely the participants were to recommend the game to friends using an 11-point Likert scale developed by Reicheld [76]. He argued that products and services were often positively evaluated through social desirability. Hence, his NPS compares the shares of promoters (two highest response options) and detractors (lowest to ninth highest response options) [76], using a score between −100% and +100%. This score can be seen as an indicator of a game’s acceptance by potential users. Figure 3 illustrates the research framework for assessing both motion-based exercise games.

### Statistical Analysis

We used 6-point Likert scales to assess all subjective measures. To increase legibility, they were rescaled to percentages (0%–100%). We used bivariate correlations (Pearson r, Spearman ρ), χ2, univariate and multivariate analyses of variance (MANOVA), and multiple linear regression for data analysis. We used and reported Pillai value V for omnibus MANOVAs. For multiple linear regressions, we used the ENTER method, iteratively removing models with low standardized beta’s, and excluded models with high variance inflation (≫1). We set the type I error rate (level of significance) to alpha=.05 and reported effect sizes as partial η^2^. The error bars in the diagrams show the 95% CI. Missing values were deleted listwise on a per test basis.

To study the influence of age using factorial methods, we split both samples at the age median in a younger and older half. This can pool individuals together who may differ greatly with regard to attitudes and behaviors, current life situation, or individual aging. In addition, this approach increases the comprehensibility of the findings and is admissible. Correlation analyses with age as ordinal value supported all subsequent analyses.

### Description of the Samples

#### Sample 1

Participants of the first study were recruited in an orthopedic practice in Germany. As the study has a usability focus, neither a medical indication nor a special effect was instructed. The sample comprised 71 participants (35 males, 49%; 36 females, 51%) age range 20 to 86 years (mean 48.4, SD 21.9). In all, 28% (20/71) participants reported a chronic illness, such as diabetes, asthma and allergies, hypertension, and/or back problems. Contrary to our expectations, age and chronic illnesses were unrelated in this sample (ρ=0.230, *P*=.06). The reported pain before the intervention appeared related to age (*r*=0.371, *P*=.002) and reported chronic illnesses (ρ=0.346, *P*=.03). Older people and people with chronic illnesses initially felt greater pain.

Age was neither related to SET (*r*=−0.223, *P*=.06) nor related to NfA (*r*=−0.135, *P*=.26), but it was linked to the reported GF (*r*=−0.591, *P*<.001). Hence, despite the scale’s variety in different games, older people reported playing less than younger people.

Gender was linked to SET (ρ=−0.431, *P*<.001), with women reporting lower self-efficacy. Self-efficacy was neither related to GF (ρ=−0.151, *P*=.20) nor related to NfA (ρ=−0.172, *P*=.15). See Multimedia Appendix 3 for a summary of the sample’s characteristics.

#### Sample 2

The second study took place in the AAL lab. A total of 64 volunteers (32 male, 32 female, 50% each), with age range 17 to 85 years (mean 43.2, SD 19.6), participated in the second study. None of the participants were part of the first study.

Similar to the first sample, 26% (17/64) participants reported a chronic illness (mainly asthma, hypertension, and diabetes), and the prevalence of chronic illnesses appeared to increase with age (ρ=0.406, *P*=.001). Reported pain before the game increased with age (*r*=0.425, *P*<.001) and chronic illnesses (ρ=0.343, *P*=.006).

Age was linked to lower SET (*r*=−0.548, *P*<.001), lower GF (*r*=0.569, *P*<.001), but it was unrelated to NfA (*r*=−0.145, *P*=.25). Also, there was a significant correlation between the participants’ gender and SET (ρ*=*−0.331, *P*=.007), with women reporting lower self-efficacy. Multimedia Appendix 3 shows the characteristics of the second sample.

## Results

### Overview

First, we analyzed performance in the game, how the performance evolved over the course of the game, and if user factors influenced attained performance. Second, we studied the games’ effect on pain and perceived exertion. Finally, we presented results from the overall usability and acceptance evaluation of the games. Owing to congruence between the evaluations, the results for both games are presented directly one after the other.

### Performance

**Figure 4:**
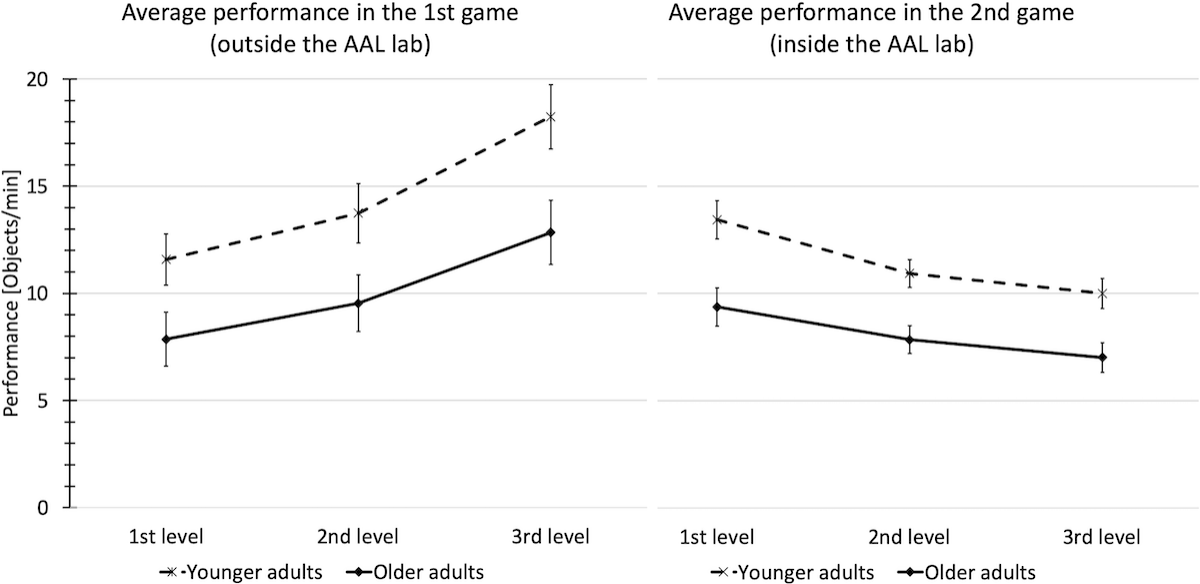
The influence of age on performance in both games across the three investigated levels. Left: Outside the ambient assisted living (AAL) environment. Right: Inside the AAL environment. Error bars indicate the 95% CI.

In the first game, the players collected on average 9.7 (SD 4.1) objects per minute in the first level, 11.7 (SD 4.5) in the second, and 15.5 (SD 5.1) in the third and last level of the game. In the second game, players collected an average of 11.4 (SD 3.2) objects per minute in the first level, 9.4 (SD 2.4) in the second, and 8.5 (SD 2.5) in the third level (see Figure 4).

To understand if user factors relate to performance, we first calculated a single average performance score across the three levels for each player.

For both samples, correlation analyses identified age as the strongest predictor for performance (*r*=−0.564 and *r*=−0.710, respectively) with older adults being slower than younger adults. Two further consistent findings were the strong positive influence of SET (*r*=0.489 and *r*=0.562, respectively) and of prior GF (*r*=0.459 and *r*=0.552, respectively). People reporting higher technical competency and higher GF were faster. The influence of NfA was only significant in the second (*r*=0.314) but not in the first game (*r*=0.227, *P*=.06).

Performance was not affected by gender (ρ=0.184, *P*=.12) or the presence of a chronic illness (ρ=0.059, *P*=.63) in the first game, although it was in the second (ρ=−0.354 and ρ=−0.38, respectively), with women and the chronically ill being slower. Table 1 summarizes these findings.

**Table 1:** Significant correlations between user factors and average performance.

Factor	Performance in game 1	Performance in game 2
Age	−0.564	−0.710
Gender	—^a^	−0.354
Self-efficacy in interacting with technology	0.489	0.562
Need for achievement	0.227	0.314
Gaming frequency	0.459	0.552
Chronic illness	—	−0.382

^a^A value was calculated but not presented due to missing significance.

To identify which of the correlating variables had the strongest influence on performance, we then calculated two multiple linear regressions with average performance as dependent variable and the user factors as predictors. The regression models were significant for the first (*F*_6,61_=10.446, *P*<.001) and second games (*F*_6,56_=16.322, *P*<.001) and explained over 50.1% (r^2^=0.507*)* and 66.2% (r^2^=0.662) of the variance in performance, respectively.

For both games, age remained the strongest predictor of performance (beta=−.521 and beta=−.608, respectively), and gender was not significant in the first model (beta=−.182, *P*=.07), although it was in the second model (beta=−.271, *P*=.003). The last remaining predictor of performance in the second game was NfA (beta=.248). Tables 2 and 3 show the coefficients from the regression models. The prior influence of SET, GF, and chronic illnesses (identified by the correlation analyses), disappeared in the regression models that controlled for the other variables.

**Table 2:** Regression table for the dependent variable performance in the first game (outside the ambient assisted living environment; n=71; r^2^=0.507; variance inflation ≤1.927).

Factor	B	SE	Beta	*t* value (*df*=65)	*P* value
(Constant)	19.534	3.823	N/A^a^	5.110	<.001
Age (years)	−0.102	0.024	−.521	−4.172	<.001
Gender	−1.501	0.807	−.182	−1.860	.07
Chronic illness	0.697	0.897	.073	0.777	.44
Self-efficacy in interacting with technology	0.314	0.489	.072	0.642	.52
Need for achievement	−0.848	0.694	−.115	−1.223	.23
Gaming frequency	0.976	0.602	.181	1.622	.11

^a^Not applicable.

**Table 3:** Regression table for the dependent variable performance in the second game (in the ambient assisted living environment; n=64; r^2^=0.662; variance inflation ≤2.182).

Factor	B	SE	Beta	*t* value (*df*=58)	*P* value
(Constant)	25.248	3.166	N/A^a^	7.976	<.001
Age (years)	−0.157	0.031	−.608	−5.062	<.001
Gender	−2.698	0.872	−.271	−3.095	.003
Chronic illness	0.477	1.139	.041	0.419	.68
Self-efficacy in interacting with technology	0.018	0.444	.005	0.040	.97
Need for achievement	1.125	0.411	.248	2.737	.009
Gaming frequency	0.671	0.709	.109	0.947	.35

^a^Not applicable.

The high beta coefficient in the regression models showed that the influence of age on performance was strongest. An RM-MANOVA with both age groups as independent variables and performance across the levels as within-subject variable illustrates this effect. Figure 4 shows the significant effect of age group on average performance for the levels in the first (*F*_1,69_=26.028, *P*<.001; η^2^=0.277) and second games (*F*_1,62_=48.175, *P*<.001; η^2^=0.473).

### Perceived Exertion and Perceived Pain

Both games had a significant overall effect on perceived exertion and PAIN (game 1: V=0.242; *F*_2,63_=10.044, *P*<.001; η^2^=0.242; game 2: V=0.360; *F*_2,61_=17.193, *P*<.001; η^2^=0.360), and this effect interacted with age group (game 1: V=0.166; *F*_2,63_=6.282, *P*=.003; η^2^=0.166; game 2: V=0.172; *F*_2,61_=6.326, *P*=.003, η^2^=0.172), indicating a possible difference between younger and older participants.

With regard to perceived exertion, results differed between the studies. The change was different for younger and older participants in the first game (*F*_1,64_=5.010, *P*=.03; η^2^=0.073). Although exertion did not change for younger participants (26.5% to 25.6%), it reduced from 25.6% to 16.3% for older participants. A different picture emerged for the second game. Perceived exertion increased for both younger (14.4% to 19.4%) and older participants (19.4% to 24.4%; *F*_1,62_=7.215, *P*=.009; η^2^=0.104).

**Figure 5:**
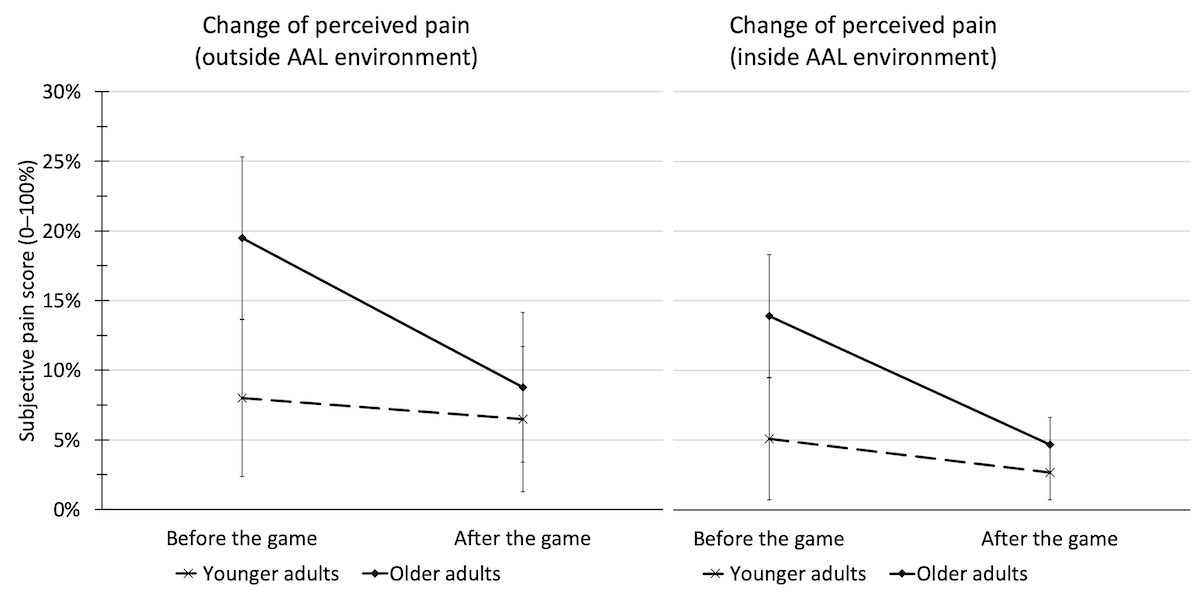
Pain-mitigating effect of both exercise games (left: outside AAL lab; right: inside AAL lab) before and after the game for younger and older participants. Error bars indicate the 95% CI. AAL: ambient assisted living.

The analysis of the reported pain levels revealed a pain-mitigating effect for both games and both age groups. In the first game, reported pain levels of younger participants decreased slightly from 8.0% to 6.5%, whereas the pain levels of older participants almost halved (19.5% to 8.8%; *F*_1,64_=10.570, *P*=.002; η^2^=0.142). In the second game, the pain levels of younger players decreased from the already low 5.1% to 2.7% and those of older adults fell to a *third* from 13.9% to 4.7% (*F*_1,62_=6.322, *P*=.02, η^2^=0.093). Figure 5 illustrates this pain-mitigating effect of both games for both age groups.

### Usability and Acceptance

#### Usability and Acceptance Outside of the Ambient Assisted Living Lab (Game 1)

The overall evaluation of the first game prototype was very positive. All but one participant agreed or rather agreed that controlling the game avatar using motion tracking was easy, yielding a high average evaluation (95.4%, SD 9.1%). Likewise, participants found the overall game concept (95.4%, SD 14.0%) understandable, and they reported little to no difficulties to perform the required gestures (10.8%, SD 23.3%). Consequently, the overall usability of the game was found to be high (85.8%, SD 17.7%).

The participants considered the game’s difficulty (27.2%, SD 18.5%) and the movement gestures (27.2%, SD 23.1%) as a little too easy (a score of 50% meant a balanced difficulty). Also, the reported motivation to exercise more often using the game was just slightly above the center of the scale (68.6%, SD 33.7%). Nevertheless, they reported fun and enjoyment playing the game (86.8%, SD 21.0%) and most expressed desire to play the game again (72.6%, SD 30.5%).

**Figure 6:**
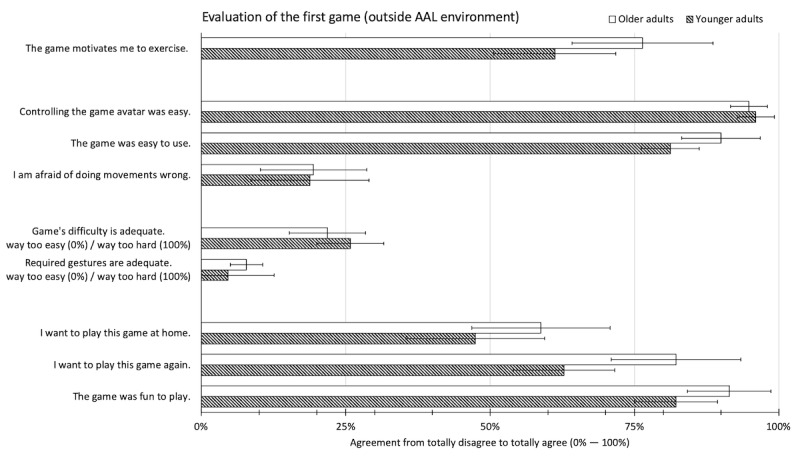
Usability evaluation of the first game prototype (outside the ambient assisted living environment) by age group. Error bars indicate the 95% CI.

Figure 6 illustrates that these findings were largely similar for younger and older adults. Significant differences were found for the desire to play the game again (*F*_1,68_=7.808, *P*=.007) and the overall usability evaluation of the game (*F*_1,68_=4.584, *P*=.04). Both greater in older adults.

Finally, we analyzed Reichheld NPS [76] and found that the majority of participants indicated they would recommend the game to others (the two topmost answers are considered as *promoters*, n=34, 48%), whereas about a quarter were neutral (n=19, 27%), and another quarter would rather not recommend the game (lowest 6 answers on the 10-point scale, n=18, 25%). Consequently, the game achieved a positive NPS of 30% (scale range from −100% to +100%).

#### Usability and Acceptance Within the Ambient Assisted Living Lab (Game 2)

**Figure 7:**
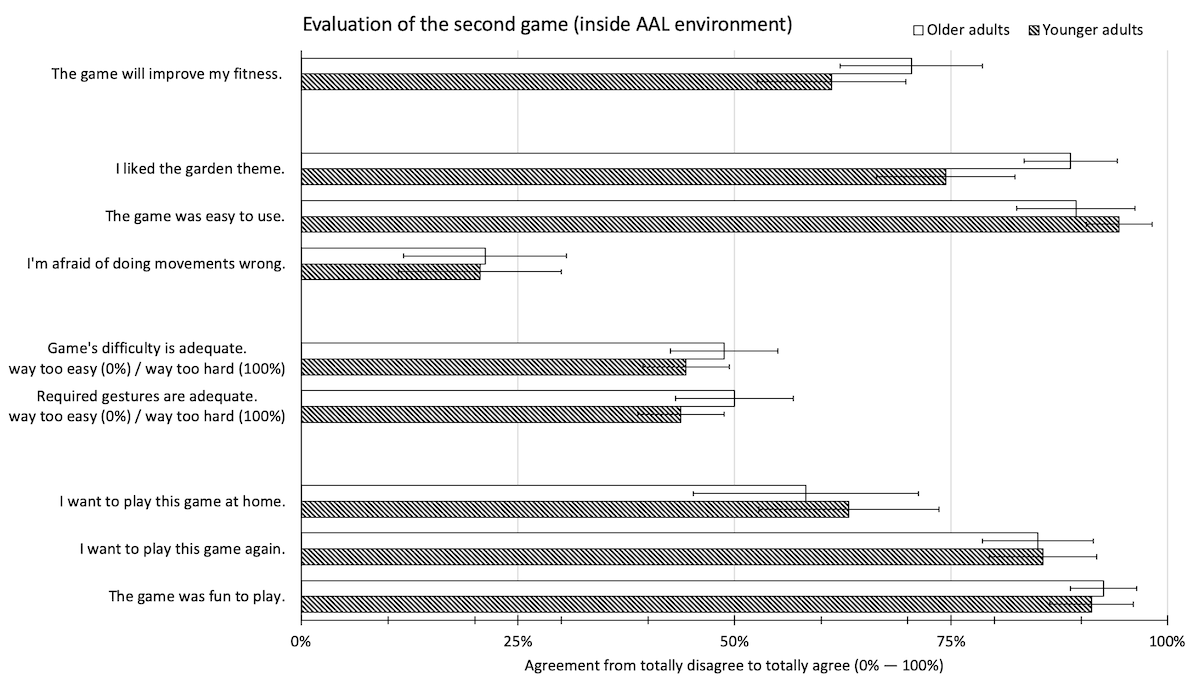
Usability evaluation of the second game prototype (in the ambient assisted living environment) by age group. Error bars indicate the 95% CI.

The participants of the second study also evaluated the game positively. Most found the game easy to use (91.8%, SD 15.4%) and reported fun playing the game (91.8%, SD 12.2%). However, despite a high desire to play the game again (85.4%, SD 17.2%), the average desire of the participants to play this game in their own home was just slightly above the center of the scale (60.6%, SD 32.8%). In contrast to the first game, the difficulty of the game as a whole (46.6%, SD 16.0%) and the difficulty of the movement gestures, in particular, were perceived as balanced (46.8%, SD 16.8%, 50% indicates a balanced evaluation). Also, the participants found the game motivating to complete exercise (86.7%, SD 14.9%). Figure 7 illustrates the results.

Finally, the NPS of the second game reached 52.6% and was therefore slightly higher than for the first game. Specifically, out of 65 participants, 29 (46%) were counted as promoters, 25 (40%) as neutral, and 9 (14%) as detractors.

## Discussion

### Principal Findings

This paper presents two serious motion-based exercise games for older adults. The stand-alone application was evaluated in the office of an orthopedic practitioner. The second game was seamlessly integrated into a prototypical AAL environment. The evaluation of both games revealed both expected and unexpected results.

First, in addition to presenting serious motion-based exercise games, we demonstrated that these games could be integrated into future AAL environments with invisibly integrated sensors and actuators to interact with the game. Consequently, the residents of technology-augmented habitats might be able to use exercise games without requiring additional hardware, visible technology, or complex setup routines, which may—if integrated into their daily routine—result in a positive effect on their physical health.

Second, we learned that user diversity is essential when designing technology for older adults or people in need of care. In our case, SET was lower for older people in both studies, which may pose a significant barrier to successful interaction with and adoption of the technology. In the present case, performance appeared to be shaped by factors we investigated. At first sight, the correlation analysis showed that performance in the exercise games was linked to age, gender (women being slower), SET, NfA, and also prior GF. After controlling for covariances, age, gender, and NfA remained strong predictors. Nevertheless, the study revealed that performance does not determine overall intention to use the game in the future. Players may be slower or faster in the game, but this appears to have had no impact on the overall acceptance and the motivation to use the game.

Third, evaluation of the games’ usability and social acceptance was not as clear as expected. In general, the evaluations of both games were positive, not only as indicated by the positive NPS but also by other metrics, such as the very high level of fun reported in the game and the high desire to play the game again. Independent of age, the players had little to no difficulties understanding the game concept or to successfully control the in-game avatar through the motion gestures captured by the Microsoft Kinect sensor. However, the participants reported a limited desire to use these motion-based exercise games in their very own home environment. We found, however, that the evaluation scores for the second game were higher than for the first. This finding suggests that a seamless integration of exercise games in the habitat might be preferred to currently existing gaming technologies that require additional hardware. Age played a role regarding social acceptance of the game, with older adults more open to the treatment. They perceived the game to be more fun and more motivating than younger adults. This might have been because of the fact that the game was specifically targeted at older adults. It is of concern, however, that prior GF is much lower for older adults. This would suggest that motion-based exercise games might be a suitable health-promoting intervention only for people who are already inclined to playing games. This, in turn, would require designing alternative health-promoting interventions that do not build on games but rather pleasant activities such as making music or art.

Fourth, we found that the perceived exertion (measured immediately before and after the game) changed. The change was only in line with expectations for the second game but not for the first. In the first game (outside the AAL environment), playing the game decreased perceived exertion in older adults, whereas it increased for both younger and older adults in the second game prototype (within the AAL environment). We speculate that this relates to the flow theory that postulates a required balance between challenge and skill [77]. The first game was found to be too easy and little challenging. Thus, people put some effort in it but probably not enough to influence exertion in younger players. In contrast, the second game was evaluated as well balanced. Accordingly, it required more effort to perform well, which yielded higher physical activity and higher perceived exertion for both younger and older players.

Fifth, the most remarkable finding was the decrease in PAIN, especially for older adults. The contrast is likely explainable as a ceiling effect given that reported pain levels before the intervention were higher for older participants than for younger participants, leaving little room for improvement. The reduction in PAIN is both astonishing and promising as it suggests that serious games may yield positive effects beyond those of increased physical fitness, physiological health, cognitive performance, and overall life expectancy as argued above [30,39,42]. Of course, a singular short-term intervention as part of a usability study should not be overinterpreted as having direct and long-lasting effects on health and pain. We rather think that the sharp pain mitigation is linked to Melzack and Wall’s theory [78] that pain perception is subject to cognitive control and can be altered. Interacting with the exercise games was perceived as fun, entertaining, and distracting, which then—presumably—lowers PAIN in older adults. This suggests that serious exercise games can yield direct positive effects on physiological health though the benefits of exercise but potentially also on overall subjective well-being by engaging in a distracting, pleasant activity. However, future work should carefully investigate if the measured effect is derived from some therapeutic utility of the game on well-being or if this is rather a placebo effect. Even if these findings were because of a placebo effect, it still follows that it is important to provide physical activity avenues for older adults that are fun and motivating.

### Future Research Questions

There is a variety of research questions that should be addressed in future work. We broadly identified and categorized six research areas.

#### Diversity of Gamers and Nongamers

While our game prototypes were in general evaluated very well, we found that prior GF influenced acceptance. Consequently, we have designed suitable exercise interventions that require physical activity but do not build on games as a persuasive, motivating element. It is also important to consider the large diversity in age, age-related changes in agility and flexibility, self-efficacy in exercising and SET, NfA, and presumably many other user factors. As such, guidelines for the design of motion-based exercise games in the sense of Gerling et al [17] should be continuously maintained and extended.

#### Variety of Game Designs

In our evaluations, we studied single-player motion-based exercise games in a garden environment. First, we need to explore many other game scenarios that might be more motivating for some players. In addition, exercise motivation also stems from social interactions and the strong power of personal social networks [79,80]. Consequently, serious exercise games should also include options for multiplayer interaction. In particular, we propose to study different types of multiplayer games on the two computer-supported cooperative work dimensions *collocated* vs *remote* and *collaborative* vs *cooperative play*.

#### Adaptive Game Environments

Although both of our games offered a limited form of adaptability (the game environment was rescaled to the players’ height), current technology offers many other exciting opportunities to tailor the game experience to a variety of different users. For example, current sensor hardware enables us to detect the current pulse (as an indicator for exertion) from a distance. It is quite possible to build a balanced feedback loop that—within medically sound ranges—continuously adjusts the games’ difficulty to the players’ exertion. Also, the games might offer specific exercise targets to reflect individual training or the rehabilitation needs of the players.

#### Long-Term Effects and Transfer

The proposed health benefits of our games have not yet been formally shown but are deduced from related work. Future work must evaluate the long-term adherence and long-term effect of using these games, especially if they are embedded in technology-augmented habitats. In addition, the prototypes evaluated here showed pain-mitigating effects. Besides the proposed benefits on physiological health and well-being, research suggests that cognitive performance increases after 30 min of physical exercise [81] (see Background). This merits further research into how cognitive performance and executive functioning improve through the long-term and regular use of motion-based exercise games.

#### Incentives

This work suggests the benefits of exercise for individuals and for society as a whole (see Background). The societal benefits touch the ethical question of if and how the use of exercise games for prevention or rehabilitation can or should be incentivized beyond the persuasive power of the games itself. It should therefore be discussed if insurance or health care institutions can or should offer rewards for using these games or if nonusage can or should be penalized.

#### Privacy

Using digital systems leaves individual digital footprints that might be interesting for the individual and various stakeholders. As such, future work should address whether the players’ activities can or should be tracked, stored, or shared and for how long. A discussion should also follow about data access, especially in relation to medical personnel and/or insurance carriers.

### Limitations

This study is not without its limitations: First, the usability and acceptance evaluation and the measured effect on PAIN and exertion is based on a short-term intervention involving approximately 10 min of gameplay. Future work should evaluate if and for how long the positive effects on well-being persist, whether and by whom the games will be used over longer periods, and whether and to what extent health improves. Second, we frequently contrasted the effect of age on performance in and perception of the game using two age groups. However, pragmatically someone does not become old by virtue of crossing the arbitrary threshold of the median splits. Also, age and aging are more complex than just considering the biological age. We should therefore intensify the investigation of individual cognitive and physical changes on the performance, acceptance, and use of serious games by increasing sample size and user diversity.

### Conclusions

The study shows that the serious exercise games developed can represent one approach to meet the challenges of demographic change and contribute to individuals’ health and well-being. Due to the user-centered and participatory development approach, both games are usable and accepted by older people. The integration into AAL environments is possible and promises an easy integration of the games into the daily routine. A flabbergasting result of the studies is the indication of a pain-reducing effect of the games for older people, which, however, needs further investigation.

## Appendix

Multimedia Appendix 1 Illustration of the user-centered and participatory design process.

Multimedia Appendix 2 Movement gestures in the games.

Multimedia Appendix 3 Characteristics of the samples.

## Abbreviations

AAL: ambient assisted living
GF: gaming frequency
MANOVA: multivariate analyses of variance
NfA: need for achievement
NPS: net promoter score
PAIN: perceived pain
SET: self-efficacy in interacting with technology
