# Supplementary figures and images for: Serious Motion-Based Exercise Games for Older Adults: Evaluation of Usability, Performance, and Pain Mitigation
Source: JMIR Serious Games. 2020 Apr 1;8(2):e14182. doi: 10.2196/14182 (PMC7160710; doi:10.2196/14182)

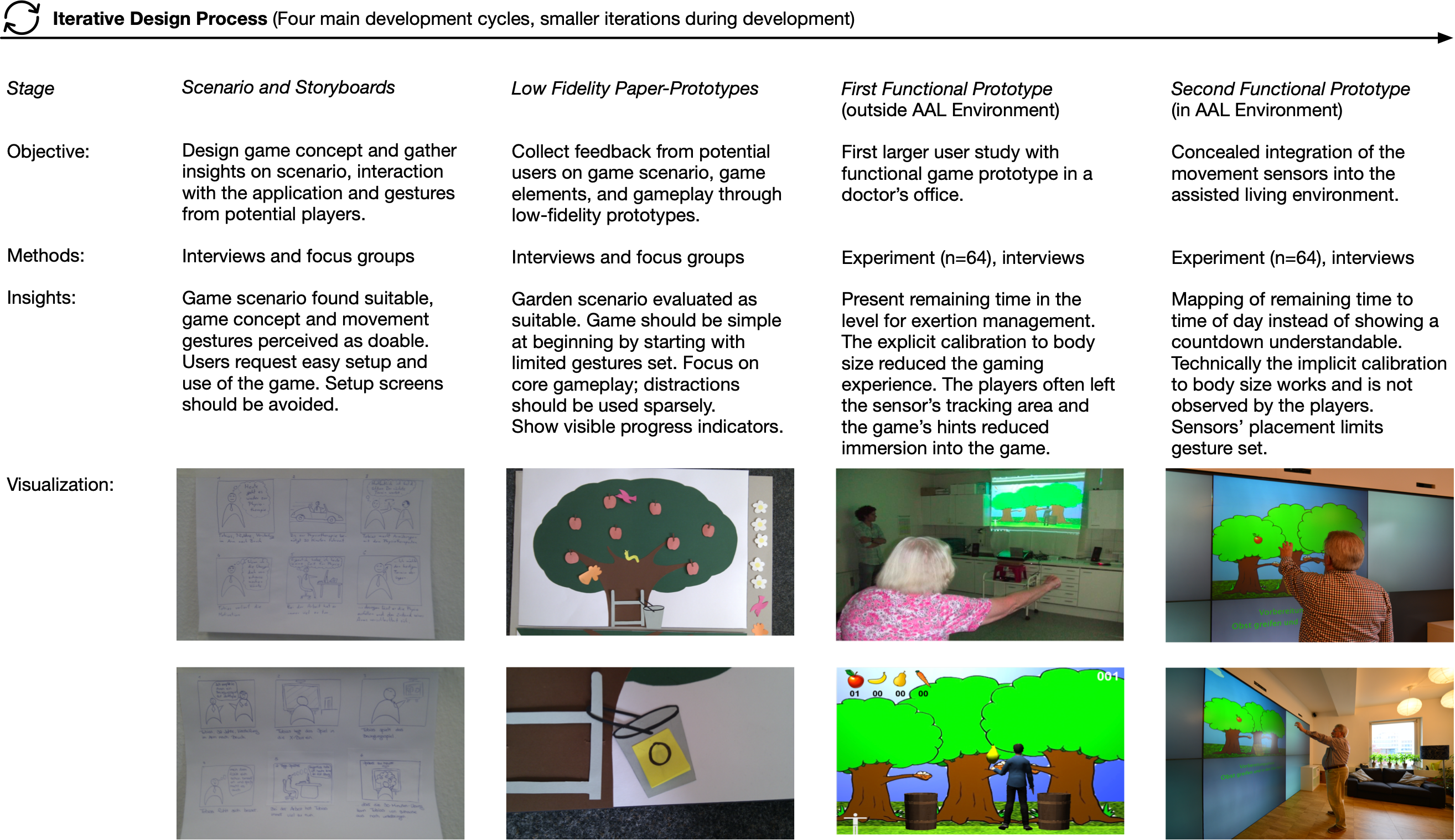

Supplement: Multimedia Appendix 1 [file games_v8i2e14182_app1.png]
