# Supplementary material for: Serious Motion-Based Exercise Games for Older Adults: Evaluation of Usability, Performance, and Pain Mitigation
Source: JMIR Serious Games. 2020 Apr 1;8(2):e14182. doi: 10.2196/14182 (PMC7160710; doi:10.2196/14182)
Supplement: Multimedia Appendix 2 [file games_v8i2e14182_app2.pdf]

Gestures used in the three levels of the games.

Level	Game 1 (outside AAL)	Game 2 (inside AAL)
1	Apples: They appear in the tree and can be collected by touching them with any hand (left or right).	Apples: They appear in the tree and can be collected by touching them with any hand (left or right).
2	Apples and Carrots: Carrots appear at the ground and require bending.	Apples and Bananas: Bananas are collected by moving hand to target and holding the position for a short time (500ms) to maintain muscle tension.
3	Apples, Carrots, and Bananas: Bananas: Collected by moving hand to target and holding the position for a short time (500ms) to maintain muscle tension.	Apples, Bananas, and Pears: Pears require diagonal movement (object left/right of the player must be picked with the right/left hand)
