# Supplementary material for: Serious Motion-Based Exercise Games for Older Adults: Evaluation of Usability, Performance, and Pain Mitigation
Source: JMIR Serious Games. 2020 Apr 1;8(2):e14182. doi: 10.2196/14182 (PMC7160710; doi:10.2196/14182)
Supplement: Multimedia Appendix 3 [file games_v8i2e14182_app3.pdf]

Characteristics of the first sample of the motion-based exercise game evaluated outside the living lab (gender dummy coded as male=1, female=2; ** p<.001, * p<.05, () p<.1)

| | **Descriptives** | **2** | **3** | **4** | **5** | **6** | **7** |
|---|---|---|---|---|---|---|---|
| 1. Age | 48.4±21.9 (20–86 years) | | -.521** | | -.591** | .371** | (.227) |
| 2. Gender (ρ) | 35 male, 36 female | — | -.252* | | | | |
| 3. SET | 3.31±0.71 | | — | | .396** | -.303* | |
| 4. NfA | 2.80±1.05 | | | — | | | |
| 5. Gaming Frequency | 1.55±1.55 | | | | — | | -.247* |
| 6. Pain Pre | 13.7±17.2 | | | | | — | .391** |
| 7. Chronic Illness (ρ) | 19/69 | | | | | | — |

Characteristics of the second sample of the evaluation of the motion-based exercise game in the living lab (gender dummy coded as male=1, female=2; ** p<.001, * p<.05, () p<.1).

| | **Descriptives** | **2** | **3** | **4** | **5** | **6** | **7** |
|---|---|---|---|---|---|---|---|
| 1 .Age | 43.2±19.6 (17–85 years) | | -.548** | | -.553** | .425** | .406** |
| 2. Gender (ρ) | 32 male, 32 female | — | -.265* | | | | (.221) |
| 3. SET | 3.73±1.34 | | — | .393* | .623** | -.249* | |
| 4. NfA | 3.03±0.95 | | | — | | | |
| 5. Gaming Frequency | 1.72±0.82 | | | | — | | |
| 6 .Pain Pre | 9.4±13.1 | | | | | — | .313* |
| 7. Chronic Illness (ρ) | | | | | | | — |
